# Effects of Acoustic Paired Associative Stimulation on Late Auditory Evoked Potentials

**DOI:** 10.1007/s10548-018-00695-4

**Published:** 2018-12-24

**Authors:** Robert Markewitz, Sarah Engel, Berthold Langguth, Martin Schecklmann

**Affiliations:** Department of Psychiatry and Psychotherapy, University of Regensburg, Universitätsstraße 84, 93053 Regensburg, Germany

## Abstract

Paired associative stimulation (PAS), a form of non-invasive cortical stimulation pairing transcranial magnetic stimulation (TMS) with a peripheral sensory stimulus, has been shown to induce neuroplastic effects in the human motor, somatosensory and auditory cortex. The current study investigated the effects of acoustic PAS on late auditory evoked potentials (LAEP) and the influence of tone duration and placebo stimulation. In two experiments, 18 participants underwent a PAS with a 4 kHz paired tone of 400 ms duration using 200 pairs of stimuli (TMS-pulse over the left auditory cortex 45 ms after tone-onset) presented at 0.1 Hz. In Experiment 1 this protocol was contrasted with a protocol using a short paired tone of 23 ms duration (PAS-23 ms vs. PAS-400 ms). In Experiment 2 this PAS protocol was contrasted with sham stimulation (PAS-400 ms-sham vs. PAS-400 ms). Before and after PAS, LAEP were recorded for tones of 4 kHz (same carrier frequency as the paired tone) and 1 kHz as control tone. In Experiment 1, there was a significant difference between LAEP amplitudes of the 4 kHz tone after PAS-23 ms and PAS-400 ms with higher LAEP amplitudes after PAS-23 ms. Before both conditions, no difference could be detected. In Experiment 2 we observed a significant overall decrease in LAEP amplitudes pre to post PAS. Unspecific decreases of LAEP following PAS with a long paired tone (PAS-400 ms) might be related to habituation effects due to repeated presentation of sound stimuli which are not evident for PAS with a short paired tone (PAS-23 ms). Interpreting this result using the concept of temporal integration time allows us to discuss it in the context of spike-timing dependent plasticity.

## Introduction

Transcranial magnetic stimulation (TMS) can inhibit or facilitate neuronal activity via non-invasive electromagnetic stimulation. Electric currents are induced in superficial brain areas via rapidly changing magnetic fields generated by a coil of wires acting as an electromagnet (Barker et al. 1985; Di Lazzaro et al. 2004; Merton and Morton 1980; Siebner and Ziemann 2007). Applied repetitively, TMS (rTMS) induces neuroplastic changes via mechanisms of long-term potentiation (LTP) or depression (LTD) (Rossi and Rossini 2004; Thut and Pascual-Leone 2010). Depending on the frequency of the applied pulses, rTMS has inhibitory (up to 1 Hz) or facilitatory (over 1 Hz) effects on brain activity (Hallett 2007; Robertson et al. 2003; Siebner and Ziemann 2007).

Paired associative stimulation (PAS) is one specific rTMS protocol which combines direct stimulation of the brain via very low-frequency rTMS (e.g. 0.1 Hz) with a corresponding peripheral sensory stimulation (e.g. direct stimulation of the area of the somatosensory cortex representing the hand via rTMS combined with electric stimulation of the hand) (Wolters et al. 2005). The timing of peripheral and central stimulation is crucial as the assumed neuroplastic mechanism is spike timing dependent plasticity (SDTP) (Wolters et al. 2003). According to the model of STDP the synaptic strength between two neurons is enhanced if postsynaptic activity is preceded by presynaptic activity. Inversely, the link weakens if the postsynaptic neuron is activated prior to the presynaptic neuron (Markram et al. 2011). In the model of STDP the pairing needs to occur within a critical time period [“tens of milliseconds or less” (Markram et al. 1997)] in order to induce changes on synaptic strength. In addition, the closer the pairing of pre- and post-synaptic activation is, the more pronounced are the effects (Levy and Steward 1983; Markram et al. 1997; Song et al. 2000). TMS pulses are considered to induce postsynaptic activity and peripheral stimulation is associated with presynaptic activity (Tzounopoulos et al. 2004). For the exact timing between TMS and peripheral stimulation, the transition time of the stimulus from the periphery to the cortex has to be considered.

Several studies of PAS of the somatosensory and motor cortex have revealed results that concur with the concept of STDP (Stefan et al. 2000, 2002, 2004; Wolters et al. 2003, 2005). These studies have also found that the effects of PAS can be measured for up to 90 min following the intervention (Stefan et al. 2000; Wolters et al. 2003).

Besides the extensively investigated motor and somatosensory system, two PAS pilot studies of the auditory cortex were conducted so far by our workgroup (Engel et al. 2017; Schecklmann et al. 2011). In the first study by Schecklmann et al. it could be shown that inhibitory effects of PAS were associated with a close timing between the cortical arrival of a sine tone and the application of a TMS pulse as indicated by a decrease in the amplitude of the long-latency auditory evoked potentials (LAEP) N1 and P2. The N1 and P2 are part of the P1–N1–P2 complex, originating in the secondary auditory cortex with latencies of 50 ms, 100 ms and 200 ms respectively (Buettner et al. 2005; Woods et al. 1987). With a temporal gap of 5 ms between the TMS pulse (45 ms) and the onset of the N1-component (50 ms) of the LAEP, the PAS condition with an inter-stimulus interval of 45 ms between tone onset and TMS pulse proved most effective in this study.

Among several open questions, three were investigated by the subsequent work of Engel and colleagues in two experiments (Engel et al. 2017). One open question was whether the effects of auditory PAS take place on the level of the primary or secondary auditory cortex. This can be investigated by using auditory steady state responses (ASSR) (Santarelli et al. 1995) elicited by tones of different amplitude modulations (AM) as dependent variable rather than AEP. For the purposes of the current study it is sufficient to know that there is evidence that the ASSR elicited by tones with an AM of 40 Hz represents activity of the primary auditory cortex (Maurer et al. 2005) and that the ASSR of tones with an AM of 20 Hz represents activity of the secondary auditory cortex (Plourde et al. 1991; Presacco et al. 2010).

Another open question was related to the importance of the duration of the paired tone. In comparison to motor/somatosensory PAS which uses peripheral electric stimuli with a duration in the range of microseconds (Stefan et al. 2000; Wolters et al. 2003), the duration of the auditory stimulus (400 ms) in the study by Schecklmann et al. was rather long. A longer duration might focus attention on the peripheral stimulus itself which was shown to influence PAS effects in the motor cortex (Stefan et al. 2004). The duration of the paired tone was therefore varied, resulting in two conditions. The most effective protocol of the study by Schecklmann et al. (2011) (featuring an inter-stimulus interval of 45 ms and a paired tone of 400 ms duration) was tested against a protocol featuring a tone of the shortest possible length that allows for a pure sine tone (23 ms) to see if the decreases in LAEP amplitudes during the first study are related to attention processes. The authors were especially interested in the effects of pure tones (even shorter durations produce click-like sounds), having in mind a potential application of auditory PAS in the treatment of tonal tinnitus.

A third open question was whether or not the effects of the study by Schecklmann et al. are caused or at least influenced by mechanisms of habituation caused by the repetitive application of acoustic stimuli. Therefore, in another experiment, the PAS protocol derived from the study by Schecklmann et al. was contrasted with a sham condition.

In sum, Engel and colleagues found a significant sham-controlled decrease specifically in the amplitude of the 20 Hz ASSR after PAS with a paired tone of 400 ms duration (Engel et al. 2017). This was interpreted as a carrier-frequency specific inhibitory effect of auditory PAS taking place in the secondary auditory cortex. No effects of the PAS intervention were found for the amplitudes of the 40 Hz ASSR. This was explained by a possible lack of appropriate stimulation of the main generators of the 40 Hz ASSR due to the specifics of their anatomical origin and properties of the TMS stimulation (Engel et al. 2017). However, since only 40 Hz ASSR were used to measure the effects of the duration of the paired tone in this study, the influence of paired tone duration on PAS effects remains unclear.

The study by Engel et al. and the study by Schecklmann et al. differed with respect to the dependent variables which were ASSR and LAEP respectively. Based on the abovementioned conclusions, auditory PAS should induce the same sham-controlled tone specific effects for LAEP (comparable to the effects of the 20 Hz ASSR). Aim of the present work is the re-analysis of the work of Engel and colleagues (Engel et al. 2017) analyzing LAEP which were the dependent variable in the study by Schecklmann et al. According to the published LAEP and ASSR findings, we hypothesize sham-controlled reductions of LAEP induced by the PAS. Longer stimulus duration is associated with increased perception of and therefore attention to the stimulus (Debner and Jacoby 1994; Overgaard et al. 2006; Sandberg et al. 2010). Thus, we also hypothesize attention-related differences in the effects for short (PAS-23 ms) vs. long (PAS-400 ms) durations of the paired tone (larger effects for a longer duration). This hypothesis is based on the reported influence of attention on neuroplasticity in the human motor cortex induced by different non-invasive stimulatory techniques such as rTMS (Conte et al. 2007, 2008), tDCS (Antal et al. 2007), and PAS (Stefan et al. 2004). Particularly the 2004 study by Stefan et al. showed larger effects of PAS when attention is directed to the paired somatosensory stimulus.

## Methods

### Subjects

With respect to sample size calculation, two statistical contrasts were the same in the present study as in the pilot study which were based on the most effective protocol [PAS(45 ms)] of the pilot study (Schecklmann et al. 2011). For the contrast post vs. pre PAS(45 ms), the effect size was d = 1.51 and with a power of 80%, alpha of 5% and two-tailed testing this resulted in a minimum sample size of 6. For the comparison between effects on LAEP for the 4 kHz tone vs. effects on LAEP for the control tone (1 kHz) (pre-post PAS differences) the sample size was 15 based on an effect size d = 0.791. Based on the comparison with effects for both control conditions during the pilot study which served as sample size estimator for the sham condition in this experiment the minimum required sample size was n = 10 for the contrast against the 0.1 Hz control condition (d = 1.05) and n = 13 for the contrast against 1 Hz (d = 0.85).

Based on these calculations, 18 healthy students (10 female, 8 male) of the University of Regensburg participated in the current study, all of whom completed both experiments. The participants’ average age was 21.3 ± 2.4 (SD) years (range of 19–28 years). All participants were right-handed. Their intelligence quotient (IQ), as evaluated by the MWT-B-Questionnaire (a German language questionnaire testing the subjects verbal IQ) (Lehrl 2005) was 118.4 ± 11.5 (104–143). One subject, for whom German was not the native language, had to be excluded from this particular assessment. Participants’ hearing thresholds were tested prior to the experiment using a standard audiometer (Midimate 622D, Madsen Electronics, USA) for frequencies ranging from 125 Hz to 8 kHz. Hearing thresholds above 30 dB HL were an exclusion criterion. Directly prior to stimulus presentation in the first session of each experiment, the sensation level (SL) for all tones used as stimuli in the experimental procedures was measured using Adobe Audition 3.0 (Adobe Systems Inc., USA). All acoustic stimuli were presented via in-ear foam headphones at 60 dB SL (E-A-RLink Foam Ear tips for Insert Earphones, Microsonic Inc., USA).

Further exclusion criteria were any history or presence of severe physical or mental disorders. All participating subjects were informed about any risks inherent with any of the experimental procedures before the first experimental session and gave written informed consent for all procedures they underwent. The experimental procedures were in agreement with the Declaration of Helsinki and approved by the Board of Ethics of the University of Regensburg (09-001).

### General Design of the Study

The same 18 subjects were included in Experiment 1 and 2. Both experiments consisted of two different PAS conditions (Experiment 1: long vs. short paired tone duration; Experiment 2: verum vs. sham stimulation). For both experiments, PAS conditions/sessions were done with an interval of 1 week in between. The interval between Experiment 1 and 2 was about 6 months. Standard condition for both experiments was PAS with a 4 kHz sinus tone of 400 ms length which was paired with TMS pulse 45 ms after tone onset (PAS-400 ms) which was shown to be the most effective (i.e. maximal inhibition of LAEP) condition in the first auditory PAS study (Schecklmann et al. 2011). Dependent variables were late auditory evoked potentials (LAEP) which were measured before and after PAS. Before and after each PAS, LAEP were recorded for pure tones of 1 kHz and 4 kHz carrier frequency, 800 ms duration with a rise- and fall-time of 75 ms. All tones were amplitude modulated at a frequency of 40 Hz during Experiment 1. During Experiment 2, two additional tones were included in the measurement of LAEP featuring the same properties as the other two tones except for an amplitude modulation of 20 Hz. The tones were presented 70 times each in a randomized order with a randomly varying inter-stimulus interval of 2300–2800 ms at 60 dB SL. Analyses of the AM results in ASSR have been presented in a separate publication (Engel et al. 2017).

### Experiment 1

**Fig. 1:**
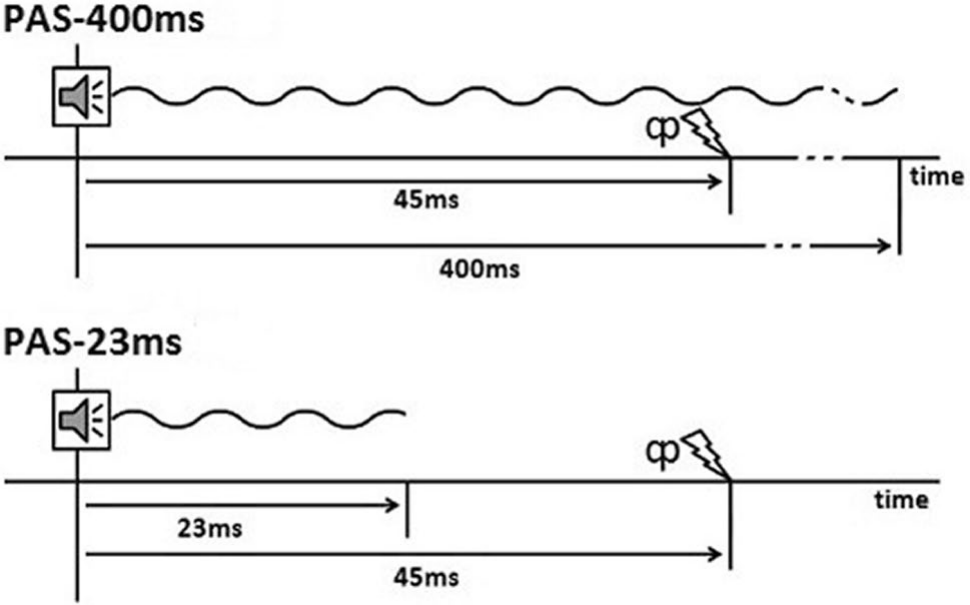
Visualization of the timing between acoustic stimulus and TMS during the PAS-400 ms (top) and the PAS-23 ms (bottom) condition

During Experiment 1, two PAS conditions varying tone duration were contrasted. Both consisted of 200 pairs of stimuli (a pure tone of 4 kHz presented binaurally, followed by a TMS pulse over the left auditory cortex 45 ms after the tone onset) presented at a frequency of 0.1 Hz. The standard condition PAS-400 ms with a tone duration of 400 ms was contrasted to a condition with a tone of 23 ms duration (PAS-23 ms). See Fig. 1 for a visualization of the timing of the different stimuli of the PAS-400 ms and the PAS-23 ms condition. The order in which each subject was presented with the two different paradigms was pseudo-randomly assigned and balanced across all participants. To avoid any possible interaction of effects of the two different paradigms, the two experimental sessions were conducted exactly one week apart. Each stimulation protocol lasted about 30 min. The LAEP recordings took approximately 7 min.

### Experiment 2

Experiment 2 contrasted PAS-400 ms with a sham stimulation (PAS-400 ms-sham). See Fig. 1 for a visualization of the timing between tone onset and TMS stimulation for the PAS-400 ms condition. For the sham condition, the back of the TMS coil was placed against the subjects’ head. This results in exposure to only a very weak magnetic field (see Section “TMS settings”) while retaining the feeling of the coil against the head of the participants as well as the clicking noise of the TMS-device whereas the tactile sensation of the TMS pulse was absent. The order in which the different paradigms were administered was pseudo-randomized and balanced across all participants with a 1-week interval between experimental sessions.

**Fig. 2:**
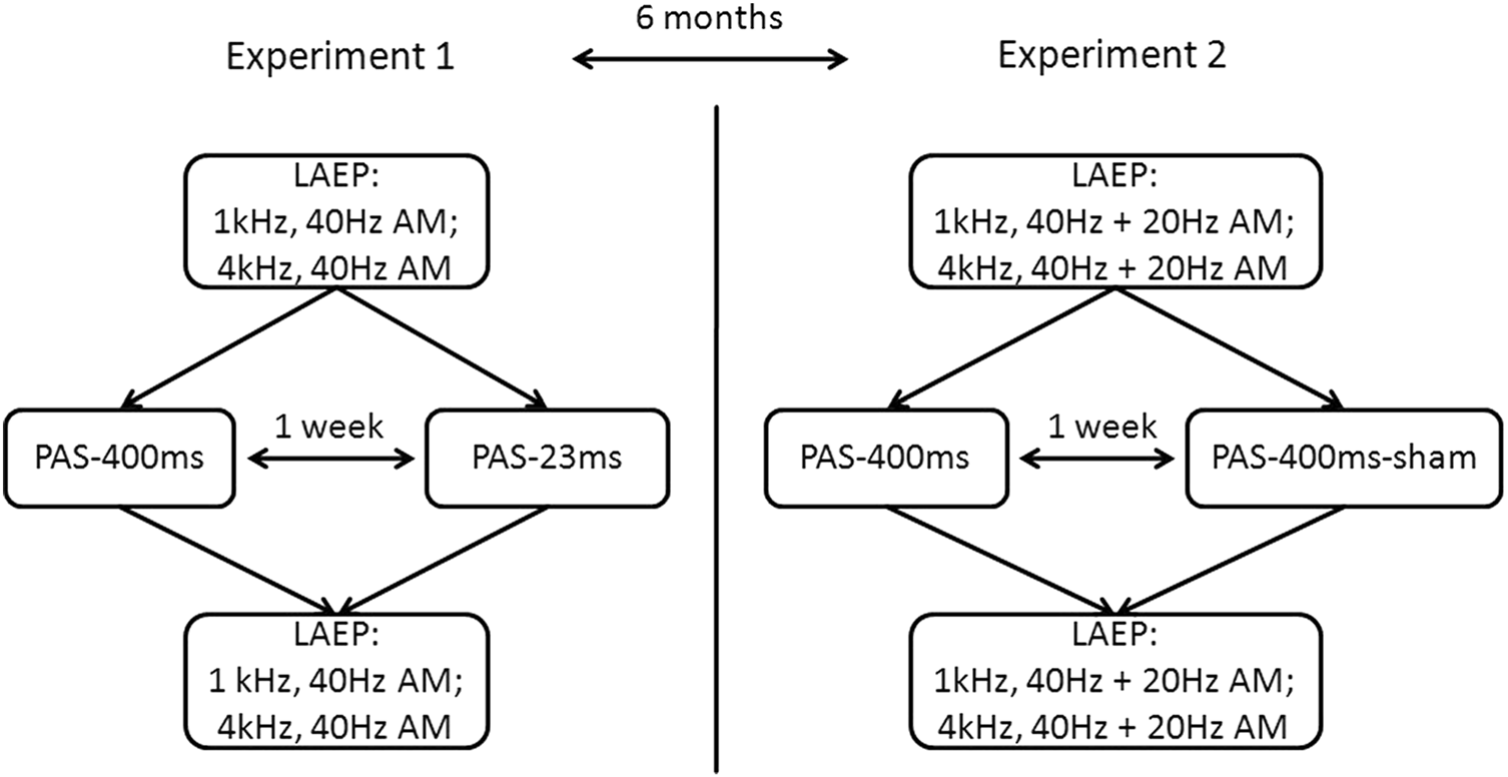
General design of Experiments 1 and 2 and intervals between experiments and experimental sessions

LAEP were recorded before and after each PAS. In this experiment, LAEP were recorded for tones of 1 kHz and 4 kHz carrier frequency amplitude modulated at 20 Hz or 40 Hz respectively. The 20 Hz amplitude modulation was introduced in order to analyze the data in the context of another study (Engel et al. 2017) with respect to 20 Hz ASSR generated primarily in the secondary auditory cortex as opposed to 40 Hz ASSR that originate mainly in the primary auditory cortex. As LAEP have neural generators in the secondary auditory cortex, it is not of relevance to differentiate between PAS effects on LAEP evoked by tones with a different amplitude modulation. For the present analysis, we averaged the LAEP of the 40 Hz and 20 Hz AM tones. Because of the inclusion of two new amplitude modulated tones in the measurement of LAEP, this step took approximately 14 min during Experiment 2. See Fig. 2 for a visualization of the overall setup of Experiments 1 and 2.

### TMS Settings

At the first session of each experiment, the subjects’ resting motor threshold (RMT) was determined following the protocol described by Pridmore et al. (1998). The term RMT describes the intensity at which a TMS-pulse over the motor cortex elicits a visually perceptible muscle twitch in five out of ten trials. The intensity of the TMS pulses during the PAS was defined to be 110% of the RMT but not more than 60% of the maximal intensity of the stimulation device. This limitation was introduced with concern to the participants’ safety and comfort. The coil position for stimulating the left auditory cortex was determined according to a standard protocol based on the international 10–20 EEG system (2.5 cm above T3 on the line between T3 and Cz and then 1.5 cm in the posterior direction perpendicular to the line T3-Cz) (Langguth et al. 2006). The same protocol was used in the 2011 study by Schecklmann et al. (2011). All experimental procedures used a water-cooled figure-of-eight coil (MAGPRO, Medtronic, USA) with a sixfold decreased magnetic field on the back side of the coil, as determined by as determined by unpublished measurements whose technical details are described in previous work (van Doren et al. 2015). This decrease in the magnetic field is of relevance for the sham condition, applied during Experiment 2.

During Experiment 1, we recorded an average RMT of 49.2 ± 4.9% of the maximal output of the TMS device. The resulting average intensity of the TMS pulses administered during PAS (110% RMT) was 53.8 ± 4.8%. The corresponding values for Experiment 2 were an RMT of 49.6 ± 5.4% and a TMS intensity of 53.8 ± 5.1%. Comparing the participants’ RMT between Experiment 1 and Experiment 2, no significant difference could be found (T = 0.414; df = 1,17; p = 0.684). Since 110% of their RMT would have exceeded 60% of the maximal intensity of the stimulation device, two subjects were stimulated with an intensity of 60% in Experiment 1 and three in Experiment 2 (due to the abovementioned concern about the participants’ safety and comfort).

### Recording and Processing of EEG Data

We used an EEG cap (62 channels) corresponding in size to the diameter of the subjects’ heads (Braincap TMS, Brain Products GmbH, Germany). The EEG cap was connected to an amplifier (Brainamp DC, Brain Products GmbH, Germany), powered by a battery (Power Pack, Brain Products GmbH, Germany). The signal was recorded with BrainVision recorder (Brain Products GmbH, Germany).

During the EEG recordings, the FCz electrode was used as reference and the AFz electrode as ground electrode. The sampling rate of the EEG was 500 Hz and the impedance level of the EEG electrodes was kept below 10 kΩ.

Preprocessing of EEG data consisted of segmentation of data from 2 s before until 2.5 s after each tone-onset. Next, the EEG data was subjected to both a high pass filter of 0.1 Hz as well as a low pass filter of 90 Hz. The segments were then visually examined for any artifacts, such as muscle contractions. Contaminated segments were manually rejected. EEG channels with low signal-to-noise ratio (no signal, 50 Hz artifacts, highly variable signal) were excluded for the next preprocessing steps. Not more than 5 channels were allowed to be excluded (in most cases it was one or two channels except for one measurement with three and one with five bad channels). Data were then assessed using an independent component analysis (ICA) to identify further artifacts, such as eye blinks, which were rejected under visual control for back-transformation of the components. Finally, all segments were re-examined for any artifacts. After artifact rejection all channels were re-referenced to an average reference allowing the reconstruction of the recording reference FCz. EEG-channels that were excluded earlier were reconstructed by interpolation of the surrounding channels. Measurements of all subjects were examined with regard to the number of segments still remaining after this step. At least 59 segments remained for all sessions. To make the measurements more comparable, only the first 59 artifact-free segments of each dataset were taken for further analysis, reducing the total number of recorded LAEP segments that were used for further interpretation from 70 to 59. Next, the segments were again filtered with a low pass filter of 18 Hz in order to eliminate all steady state responses from the data that result from stimulation with amplitude modulated tones. Processing to this point was conducted using the MATLAB (Mathworks, USA) toolbox EEGLAB (Delorme and Makeig 2004). For further analysis the data was transferred to the FieldTrip toolbox (Oostenveld et al. 2011).

The 59 trials of each measurement were averaged and baseline corrected for the interval 300 ms before the onset of the tones. The resulting data was then examined with regards to its plausibility as components of LAEP, considering the topography as well as the time course of fluctuations in the EEG.

### Identification of LAEP

**Fig. 3:**
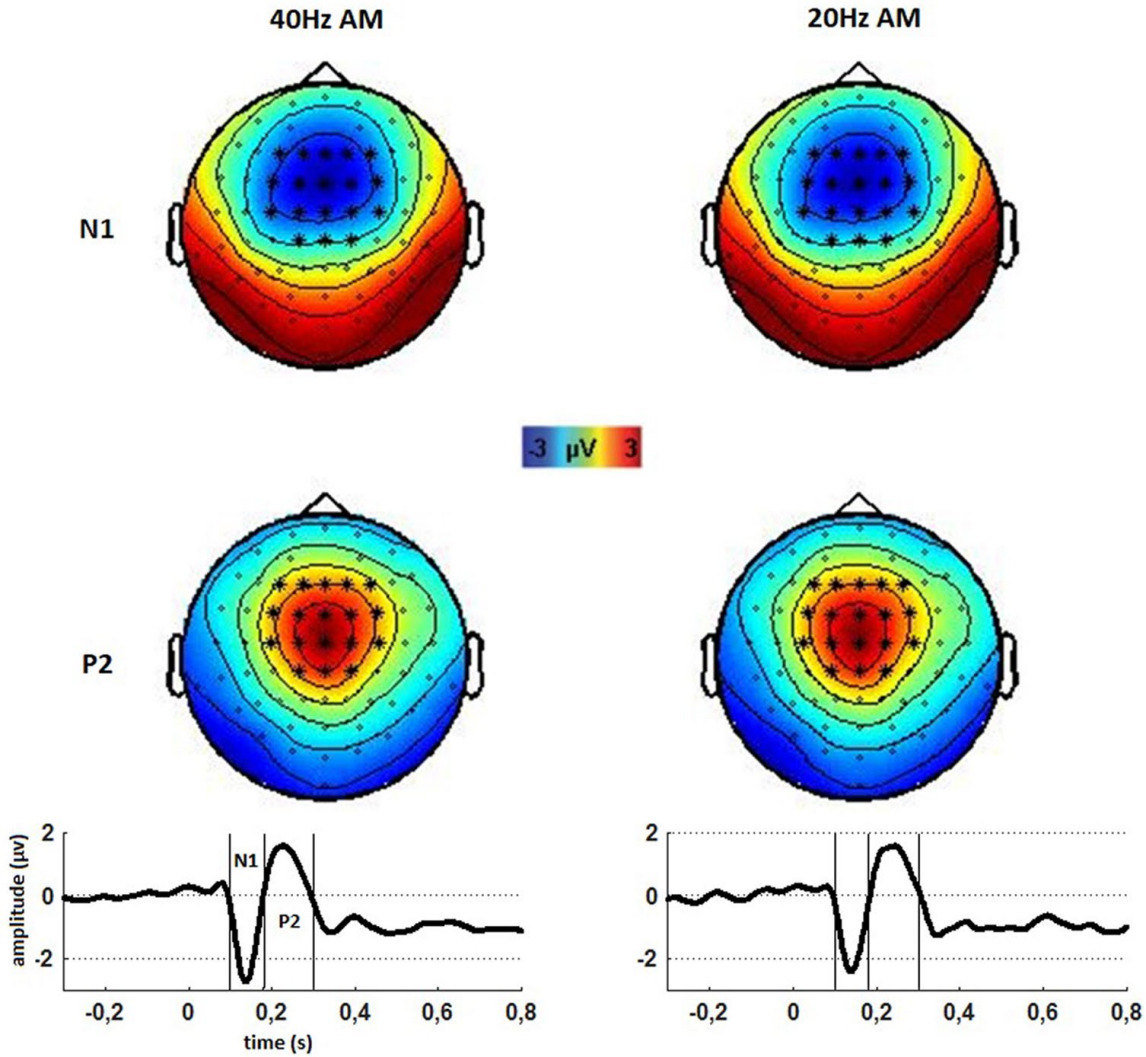
Topographies (top) of the N1–P2-complex and trajectories (bottom) of the N1 and P2 for 40 Hz (left) and 20 Hz (right) amplitude modulation tones for measurements before PAS. Asterisks and lines mark the channels and time of interest which were averaged for subsequent analyses for all conditions

For reasons of plausibility and in order to define the channels and the time of interest for data analyses, the EEG data recorded prior to the PAS was inspected with regard to the evoked activity. These pre-PAS data revealed clear auditory evoked activity as indicated by fronto-central topography and a negative deflection at 100 ms (LAEP N1) followed by a positive peak around 200 ms (LAEP P2) after tone onset. The topographies of the N1 and the P2 component for the tones of 40 Hz and 20 Hz amplitude modulation as well as the trajectories of the recorded LAEP for the 20 Hz and 40 Hz amplitude modulated tones in the region of interest can be seen in Fig. 3.

The time of interest was chosen according to the points were signals crossed the zero line which was at 100 ms and 180 ms for the N1 and at 180 ms and 300 ms for the P2. Based on these topographies of pre-PAS evoked activity, the channels of the electrodes F3, F1, Fz, F2, F4, FC3, FC1, FCz, FC2, FC4, C3, C1, Cz, C2, C4, CP1, CPz and CP2 were chosen as channels of interest for further analyses. Time and channels of interest were averaged for 20 Hz and 40 Hz AM tones as trajectories and topographies were similar and as LAEP are considered to have neural generators in the secondary auditory cortex. The N1 and the P2 components are thought to originate from neural generators in the secondary auditory cortex, with some contribution from other cortical regions (Eggermont and Ponton 2002; Woods et al. 1987). No clear P1 peak was evident which is similar to the findings of the study by Schecklmann et al. (2011). As in this study, we averaged the channels and time intervals of interest for the N1 and P2 and calculated the peak-to-peak difference of both LAEP (P2–N1) which was used as dependent variable.

### Statistical Evaluation of the EEG Data

The average of channels and time intervals of interest were exported to SPSS (IBM Corp., USA) and assessed using repeated measure analyses of variance (rm-ANOVAs). Overall rm-ANOVAs for Experiments 1 and 2 were calculated. For Experiment 1 a three-factorial rm-ANOVA was performed with the within-subject factors ‘time’ (pre PAS vs. post PAS), ‘tone duration’ of (23 ms vs. 400 ms) and ‘frequency’ (4 kHz (same carrier frequency as the paired PAS tone) vs. 1 kHz control tone). For Experiment 2, a three-factorial rm-ANOVA was calculated with the within-subject factors ‘time’ (pre PAS vs. post PAS), ‘PAS’ (active vs. sham) and ‘frequency’ (4 kHz (same carrier frequency as the paired PAS tone) vs. 1 kHz control tone). LAEP for both 20 Hz and 40 Hz AM tones in Experiment 2 were averaged (see methods and results). Overall rm-ANOVAs were performed two-tailed with a statistical threshold of 5%. For post-hoc tests we used subsequent rm-ANOVAs and t-tests using a Bonferroni adjusted significance threshold. In addition, we report effect sizes, i.e. partial eta-squared for rm-ANOVA (small effect: 0.02≤η^2^_p_ < 0.13; medium effect: 0.13≤η^2^_p_ < 0.26; large effect: η^2^_p_ > 0.26) and Cohen’s d for t-tests (small effect: 0.2 ≤d < 0.5; medium effect: 0.5 ≤d < 0.8; large effect: d > 0.8). All variables were normally distributed as indicated by non-significant Kolmogorov–Smirnov tests and thus fulfilled the assumptions of the used parametric tests.

## Results

### Experiment 1

**Fig. 4:**
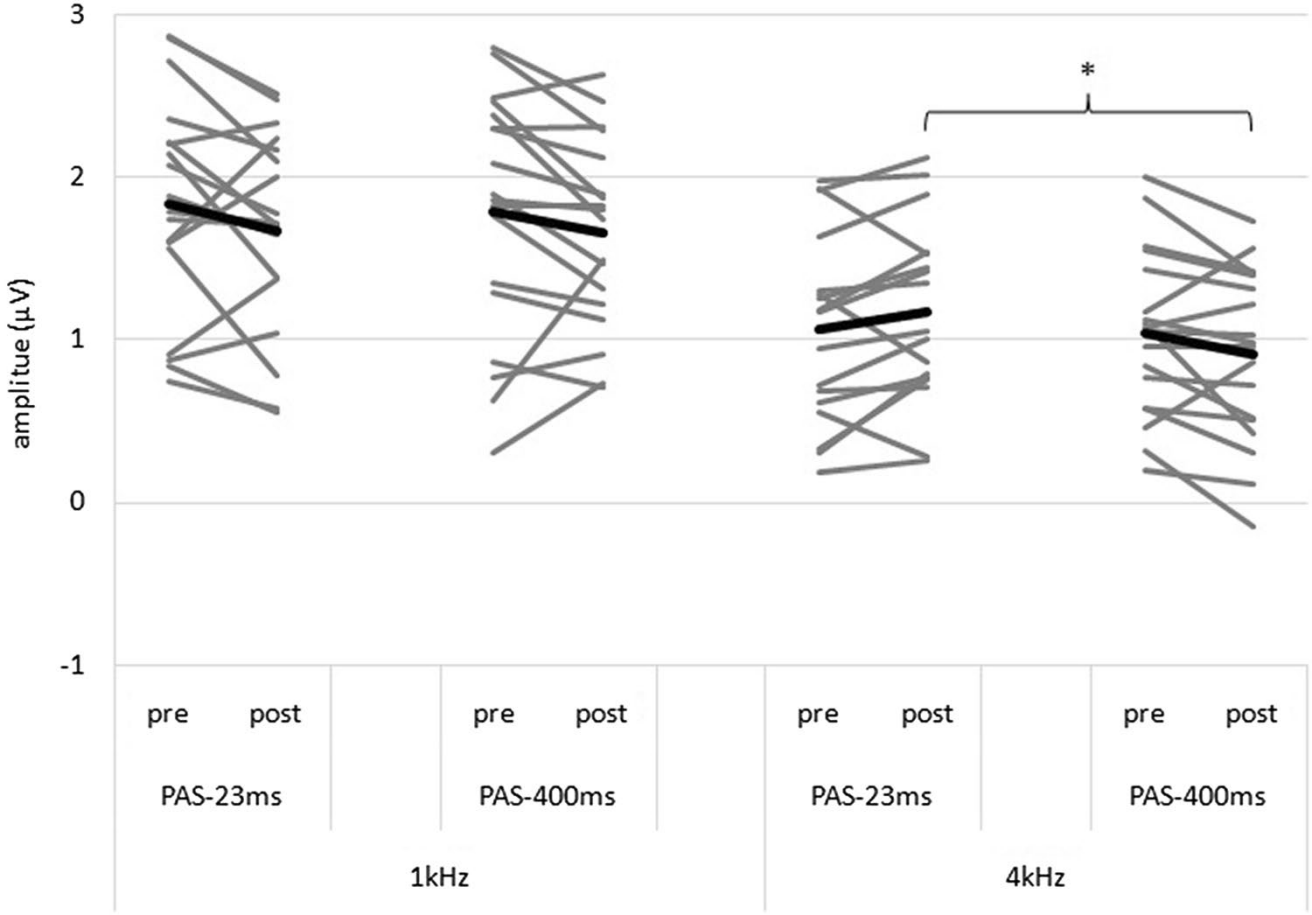
Individual changes of the N1–P2-complex from pre to post PAS for Experiment 1. Mean of each condition is indicated by the bold lines. *Bonferroni corrected statistically significant difference in LAEP amplitude between PAS-400 ms and PAS-23 ms for post PAS

The rm-ANOVA with the factors tone duration (PAS-400 ms vs. PAS-23 ms), time (pre vs. post PAS) and frequency [4 kHz (PAS paired frequency) vs. 1 kHz (control tone)] revealed a statistically significant main effect of tone frequency (F = 101.708; df = 1,17; p < 0.001; η^2^_p_ = 0.857) representing higher amplitudes for the 1 kHz in contrast to the 4 kHz tone which is a common finding (Maurer et al. 2005; Vesco et al. 1993). The main effects of tone duration and time were not statistically significant. In addition, we found a non-significant threefold interaction effect with medium effect size (F = 3.782; df = 1,17; p = 0.069; η^2^_p_ = 0.182). The non-significant interaction effects frequency by time (F = 3.562; df = 1,17; p = 0.076; η^2^_p_ = 0.173) and tone duration by time (F = 3.685; df = 1,17; p = 0.072; η^2^_p_ = 0.178) with medium effect sizes can be neglected in the light of the threefold interaction. Comparable to the study by Schecklmann et al. which also showed a non-significant overall interaction effect (p < 0.1), based on the medium effect size for the threefold interaction and a priori formulated effects of tone length we did exploratory analyses. Bonferroni corrected post-hoc rm-ANOVAs were done for both frequencies and revealed a significant duration by time interaction effect with large effect size (F = 7.795; df = 1,17; p = 0.026; η^2^_p_ = 0.314) for the 4 kHz tone but not for the 1 kHz control tone with negligible effect size (F = 0.197; df = 1,17; p > 0.999; η^2^_p_ = 0.011). Bonferroni corrected post-hoc t-tests for the 4 kHz tone revealed a significant difference with medium effect size between LAEP amplitudes after PAS-400 ms and PAS-23 ms (T = 2.849; df = 17; p = 0.044; d = 0.672) with no difference before the PAS (T = 0.279; df = 17; p > 0.999; d = 0.066) which is associated with a descriptive (statistically non-significant) increase of LAEP amplitudes after PAS-23 ms with small effect size (T = 1.762; df = 17; p = 0.384; d = 0.415) and another descriptive decrease after PAS-400 ms (T = 2.015; df = 17; p = 0.240; d = 0.475) with small effect size. For details see Fig. 4.

### Experiment 2

**Fig. 5:**
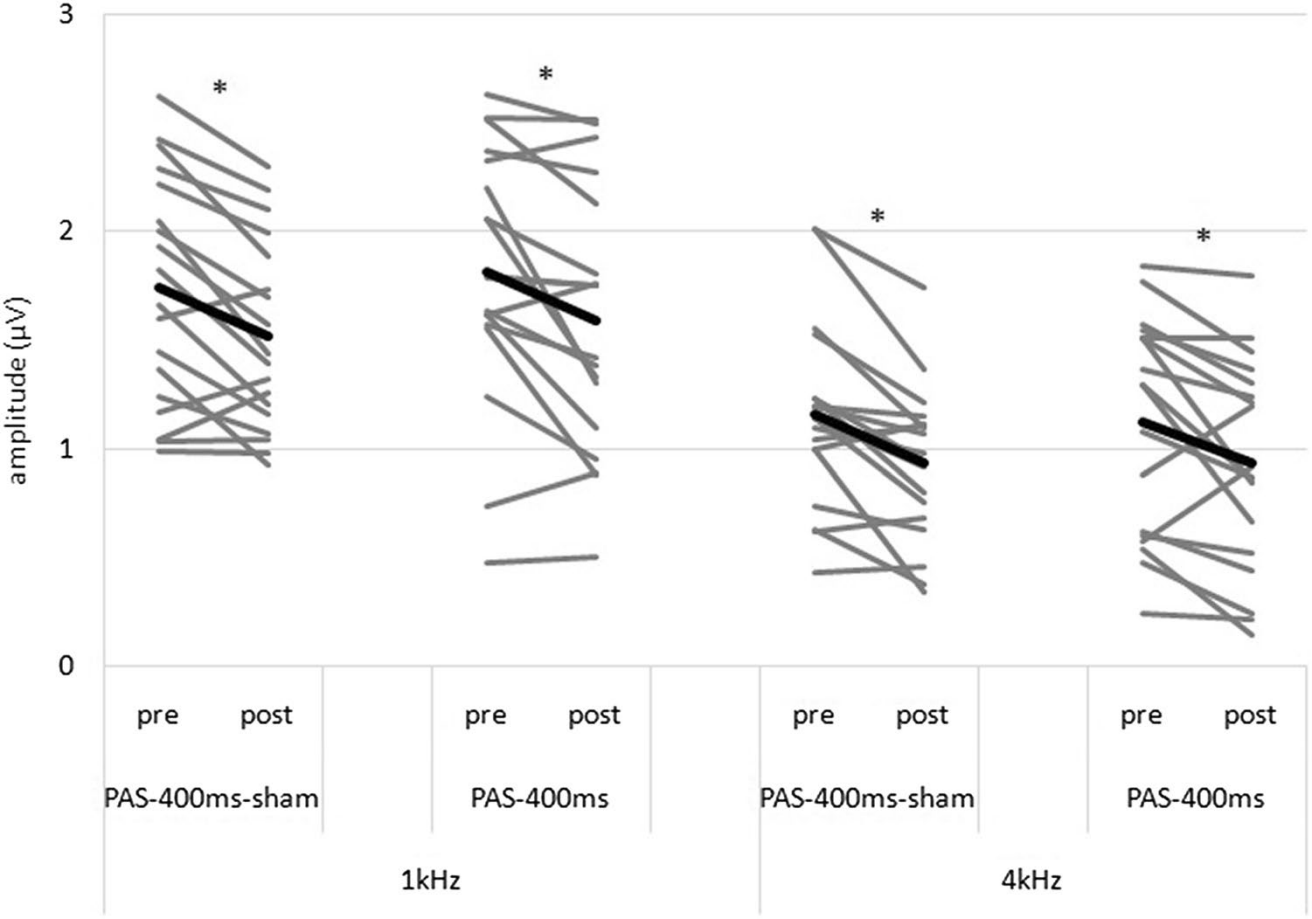
Individual changes of the N1–P2-complex for Experiment 2. Mean of each condition is indicated by the bold lines. 20 Hz and 40 Hz AM tones were averaged for each condition. *Statistically significant change in LAEP amplitude from pre to post PAS

The rm-ANOVA with the factors PAS (verum vs. sham), time (pre vs. post TMS) and frequency (4 kHz vs. 1 kHz) revealed a significant main effect of frequency with large effect size (F = 155.524; df = 1,17; p < 0.001; η^2^_p_ = 0.901) presenting again higher amplitudes for the 1 kHz in contrast to the 4 kHz tone and a significant main effect with large effect size of time (F = 2.370; df = 1,17; p < 0.001; η^2^_p_ = 0.626) which indicates a decrease of LAEP amplitudes from pre to post PAS stimulation. Importantly, this decrease occurs for all PAS conditions during experiment two, including the sham stimulation. Other main and interaction effects were not significant. For details see Fig. 5.

## Discussion

The aim of the current study was the replication of former findings of Schecklmann et al. and the re-analyses of the work of Engel et al. who both showed decreases in secondary auditory cortex activity as indicated by LAEP (Schecklmann et al. 2011) or 20 Hz ASSR (Engel et al. 2017) respectively after PAS-400 ms. We expected differences in PAS effects as a result of different durations of the paired tone [larger effects for a longer duration of the paired tone (PAS-400 ms)] and sham-controlled decreases in LAEP amplitudes.

There are two main results of this study. First, the post-PAS LAEP amplitudes of the 4 kHz tone differ significantly (large effect size) between short (PAS-23 ms) and long (PAS-400 ms) paired tones with higher LAEP amplitudes after PAS-23 ms which is mirrored by a non-significant increase (small effect size) in LAEP amplitudes from pre to post PAS-23 ms and a non-significant decrease (small effect size) from pre to post PAS-400 ms (Experiment 1).

Secondly, LAEP amplitudes decreased unspecifically from pre to post stimulation as can be seen by the significant main effect of time in Experiment 2 independent from the experimental factors which means reductions in LAEP amplitudes for the active and also the sham condition. These effects are similar in Experiment 1 but only on a descriptive level (see Figs. 4 and 5). Therefore, a long duration of the paired tone (PAS-400 ms) seems to be associated with inhibitory effects (independent of TMS). A short duration of the paired tone (PAS-23 ms) seems to have at least no inhibitory effects on LAEP amplitudes. These findings are not in line with our hypotheses. Based on the analyses of the ASSR of the same data set, as well as findings from studies of the motor cortex we expected to find sham-controlled tone specific decreases in amplitude for the LAEP as well as an influence of the duration of the paired tone with larger effects for a longer stimulus duration (PAS-400 ms). Up until now, we argued that all experimental findings of auditory PAS studies can be explained by assuming STDP as the governing mechanism of neuroplastic changes induced by PAS. In the following discussion, we try to provide possible explanations for these new, inconclusive results.

The decrease in LAEP amplitude from pre to post stimulation for both carrier frequencies (with the notable exception of the LAEP for the 1 kHz, 40 Hz AM tone) and after every PAS condition including the sham condition of Experiment 2 (and also, even if just on a descriptive level during Experiment 1—except for the LAEP for the 4 kHz tone after PAS-23 ms) may be caused by mechanisms of habituation or adaptation, as the repeated presentation of a sensory stimulus can lead to a decrease in the brains response to that stimulus (Lanting et al. 2013). According to the model of stimulus specific adaptation, processes of adaptation happen specifically for tones with the exact same features, including carrier frequency and amplitude modulation (Perez-Gonzalez and Malmierca 2014). This is in line with our findings that a decrease in LAEP amplitude happened for all tones, since the number of amplitude modulated tones of different features was exactly the same. There is evidence (Pantev et al. 1993) that LAEP are more susceptible to habituation effects than ASSR which would explain the discrepant findings between the analysis of ASSR (Engel et al. 2017) and the current LAEP findings.

Another possible mechanism might be decrease of vigilance over time for which LAEP are susceptible. For ASSR (Engel et al. 2017) it seems to be less relevant. This effect may partially explain the difference between the current results and that of the study by Schecklmann et al. (2011) in which the subjects performed a simple visual attention task instead of listening passively. The lack of experimental control for the decrease of amplitude of the LAEP is therefore a main limitation of the analysis of LAEP. Another limitation is that the sham-control was only done for PAS-400 ms (Experiment 2) and not for PAS-23 ms (Experiment 1).

Nonetheless, this decrease in LAEP amplitude was observed only for the conditions using a 400 ms long 4 kHz paired tone during PAS, whereas a decrease was not evident for the LAEP amplitudes after the PAS condition using a 23 ms long 4 kHz paired tone as mirrored by a non-significant increase with small effect size. This was found only for the LAEP evoked by a 4 kHz tone (paired with PAS) and not by the control tone of 1 kHz. This finding might be explained by the concept of “temporal integration time” (TIT) for AEP. The duration needed for a stimulus to induce particular AEP-components increases with the latency of these components. It has been suggested that for at least some of the neuronal generators of the N1 and later components, tones of a duration of at least 24 ms are needed in order for those generators to be sufficiently activated (Alain et al. 1997). This implies that the tone of 23 ms duration we used in the PAS-23 ms was too short to activate these neuronal groups reliably. A tone of a duration of 23 ms is more likely to evoke only earlier AEP-components, since the P1 components can be easily measured after short clicks used for example for P50 gating experiments (Wilde et al. 2007; Yadon et al. 2009). If, based on the concept of “TIT”, we assume that a 23 ms tone induces only AEP components of a latency of 50 ms and earlier, the majority of the neurons activated by such a short tone are stimulated before the TMS pulse that is administered 45 ms after tone onset. For these neurons, the temporal order of peripheral stimulation and TMS pulse is clearly in favor of facilitatory effects according to the model of STDP. As amplitudes of later components are dependent on effects in earlier components (Maurer et al. 2005) this effect might nonetheless be measured with LAEP. These considerations might explain the descriptive, but frequency-specific increase in LAEP amplitudes for the PAS condition using a shorter paired tone (PAS-23 ms). In this case, the induced neuroplastic effect would even be strong enough to override the unspecific inhibitory effects seen for all other conditions.

Our finding of a difference in PAS effects depending on the duration of the paired tone as well as the lack of a difference in effects for the active and the sham condition is in contrast to the ASSR findings of the same data set which showed no effects for different durations of the paired tone but sham-controlled decreases for the 20 Hz ASSR. We hypothesized that the 20 Hz ASSR specifically activates the secondary auditory cortex and does not act on the level of the primary auditory cortex (since this cortical field is located too deep within the skull for the magnetic field of the TMS to reach it) as measured by 40 Hz ASSR. Based on the superposition theory ASSR are generated by the superposition of AEP of a very specific latency/frequency. In combination with the exact timing of PAS TMS effects were specific for the 20 Hz ASSR. 20 Hz ASSR were not measured for Experiment 1 which investigated the effects of different durations of the paired tone. LAEP amplitudes may not show sham-controlled effects as the neural generation of the N1–P2 complex is not only based on auditory cortex activity covering specific PAS effects. Otherwise and as outlined above the LAEP findings reported in this study suggest effects of acoustic PAS only on AEP with latencies shorter than 50 ms which show a lower variability in their latencies when compared to AEP with longer latencies and are generated more exclusively in the auditory cortex and are thus prone to PAS effects.

In conclusion, the results of the present study seem to suggest that most of the effects on LAEP recorded after PAS-400 ms are due to unspecific mechanisms, such as habituation or adaptation, rather than to the mechanisms caused by the stimulation itself. This might, however, not be the case for PAS-23 ms. Short tone lengths of 23 ms as peripheral PAS stimuli seem to activate AEP components earlier than P1. In combination with TMS and an interstimulus interval of 45 ms auditory PAS with a short paired tone (PAS-23 ms) leads to post-PAS LAEP amplitudes that differ significantly from those resulting from auditory PAS with a long paired tone (PAS-400 ms).

However, further research is needed to examine the effects of different durations of the paired tone on the effects of PAS, possibly by comparing a wider range of different durations of the paired tone as well as investigating the effects of auditory PAS on middle latency AEP. Also, the influence of attention on the effects of auditory PAS still remains unclear since the reported difference in effects of auditory PAS with a long (PAS-400 ms) and a short paired tone (PAS-23 ms) seem to be caused by mechanisms other than attention. Therefore, future studies of auditory PAS should explore different approaches of directing attention to the paired auditory stimulus as well as their influence on PAS effects. Guiding the TMS with neuronavigation would help to answer the question at which site the assumed neuroplastic effects of acoustic PAS take place.
